# Transcriptional reprogramming during human osteoclast differentiation identifies regulators of osteoclast activity

**DOI:** 10.1038/s41413-023-00312-6

**Published:** 2024-01-24

**Authors:** Morten S. Hansen, Kaja Madsen, Maria Price, Kent Søe, Yasunori Omata, Mario M. Zaiss, Caroline M. Gorvin, Morten Frost, Alexander Rauch

**Affiliations:** 1 Molecular Endocrinology Laboratory (KMEB), Department of Endocrinology, Odense University Hospital, DK-5000 Odense C, Denmark; 2 Department of Clinical Research, Faculty of Health Sciences, University of Southern Denmark, DK-5000 Odense C, Denmark; 3 Clinical Cell Biology, Pathology Research Unit, Department of Clinical Research, University of Southern Denmark, DK-5000 Odense C, Denmark; 4 Institute of Metabolism and Systems Research (IMSR) and Centre for Diabetes, Endocrinology and Metabolism (CEDAM), University of Birmingham, Birmingham, B15 2TT UK; 5 Centre for Membrane Proteins and Receptors (COMPARE), Universities of Birmingham and Nottingham, Birmingham, B15 2TT UK; 6 Department of Molecular Medicine, University of Southern Denmark, DK-5000 Odense C, Denmark; 7 Department of Orthopedic Surgery, Faculty of Medicine, The University of Tokyo, Tokyo, 113-8655 Japan; 8 Department of Internal Medicine 3, Rheumatology and Immunology, Friedrich-Alexander-University Erlangen-Nürnberg (FAU) and Universitätsklinikum Erlangen, D-91054 Erlangen, Germany; 9 Deutsches Zentrum Immuntherapie (DZI), Friedrich-Alexander-University Erlangen-Nürnberg and Universitätsklinikum Erlangen, D-91054 Erlangen, Germany; 10 Steno Diabetes Center Odense, Odense University Hospital, DK-5000 Odense C, Denmark

**Keywords:** Bone, Osteoporosis

## Abstract

Enhanced osteoclastogenesis and osteoclast activity contribute to the development of osteoporosis, which is characterized by increased bone resorption and inadequate bone formation. As novel antiosteoporotic therapeutics are needed, understanding the genetic regulation of human osteoclastogenesis could help identify potential treatment targets. This study aimed to provide an overview of transcriptional reprogramming during human osteoclast differentiation. Osteoclasts were differentiated from CD14^+^ monocytes from eight female donors. RNA sequencing during differentiation revealed 8 980 differentially expressed genes grouped into eight temporal patterns conserved across donors. These patterns revealed distinct molecular functions associated with postmenopausal osteoporosis susceptibility genes based on RNA from iliac crest biopsies and bone mineral density SNPs. Network analyses revealed mutual dependencies between temporal expression patterns and provided insight into subtype-specific transcriptional networks. The donor-specific expression patterns revealed genes at the monocyte stage, such as filamin B (*FLNB*) and oxidized low-density lipoprotein receptor 1 (*OLR1*, encoding LOX-1), that are predictive of the resorptive activity of mature osteoclasts. The expression of differentially expressed G-protein coupled receptors was strong during osteoclast differentiation, and these receptors are associated with bone mineral density SNPs, suggesting that they play a pivotal role in osteoclast differentiation and activity. The regulatory effects of three differentially expressed G-protein coupled receptors were exemplified by in vitro pharmacological modulation of complement 5 A receptor 1 (*C5AR1*), somatostatin receptor 2 (*SSTR2*), and free fatty acid receptor 4 (*FFAR4*/GPR120). Activating C5AR1 enhanced osteoclast formation, while activating SSTR2 decreased the resorptive activity of mature osteoclasts, and activating FFAR4 decreased both the number and resorptive activity of mature osteoclasts. In conclusion, we report the occurrence of transcriptional reprogramming during human osteoclast differentiation and identified SSTR2 and FFAR4 as antiresorptive G-protein coupled receptors and FLNB and LOX-1 as potential molecular markers of osteoclast activity. These data can help future investigations identify molecular regulators of osteoclast differentiation and activity and provide the basis for novel antiosteoporotic targets.

## Introduction

Bone remodeling is a dynamic and coordinated cellular process that includes resorption of bone by osteoclasts and formation of bone by osteoblasts to maintain bone homeostasis and strength. Bone remodeling is usually balanced during early adulthood but is sensitive to changes in mechanical loading, aging, and endocrine regulators.^1^ With advancing age, an imbalance in bone remodeling caused by increased bone resorption and inadequate bone formation may lead to osteoporosis, a metabolic bone disease characterized by low bone mass and impaired bone microarchitecture, leading to increased fracture risk.

Osteoclasts are central to bone development, bone remodeling, and fracture repair. Osteoclast differentiation is a coordinated process starting with the myeloid commitment of hematopoietic stem cells to monocytes that differentiate into macrophages following macrophage colony-stimulating factor (M-CSF) signaling. M-CSF acts on its receptor, colony-stimulating factor 1 receptor (CSF1R), which induces the expression of RANK,^2^ a receptor for RANKL. RANK-RANKL binding activates pathways such as the nuclear factor κβ (NFκβ) and MAPK signaling pathway,^3,4^ among other pathways, to promote the expression of NFATc1,^5^ a master regulator of osteoclastogenesis. RANKL signaling then causes preosteoclasts to mature and fuse into multinucleated bone-resorbing osteoclasts.^6^ Notably, osteoprotegerin (OPG), which is released from stromal cells and osteoblasts, is a decoy receptor for RANKL and thereby inhibits RANKL-mediated signaling in osteoclasts.^7^ Although these regulatory mechanisms of osteoclastogenesis are well described, little is known about the temporal remodeling of the transcriptional networks that are required to turn myeloid progenitors into bone-resorbing osteoclasts.

Patients with osteoporosis exhibit increased osteoclast differentiation and activity, partly due to reduced suppression of receptor activator of nuclear κβ ligand (RANKL).^8–10^ Accordingly, the suppression of osteoclast activity is the main target for commonly used osteoporosis treatments, such as bisphosphonates or denosumab.^11^ Although current osteoporosis treatments decrease fracture risk, clinical management is limited by contraindications, adverse effects, and skeletal complications associated with long-term treatment with antiresorptive compounds, even after withdrawal.^12–15^

Therefore, there is a need to identify molecular targets that can be investigated as potential antiosteoporotic treatments. The purpose of this study was to investigate the transcriptional regulation of human osteoclastogenesis to identify undescribed regulators and predictors of human osteoclast differentiation and activity. In this study, we evaluated transcriptional reprogramming during human osteoclastogenesis using bulk RNA-sequencing (RNA-seq) at four time points throughout in vitro osteoclast differentiation. We identified 8 980 differentially expressed genes grouped into eight temporal expression profiles and highlighted the implications of stage-specific osteoclast genes in bone development, fracture repair, and the genetics of human osteoporosis. Network analyses revealed the temporal complexity and dependencies of transcriptional remodeling during human osteoclast differentiation, revealed the transcriptional networks of osteoclast subpopulations, and predicted transcription factors essential for the regulation of osteoclast genes through changes in post-transcriptional activity. Finally, to identify novel targets that can regulate osteoclast activity, we identified filamin B (*FLNB1*) and oxidized low-density lipoprotein receptor 1 (*OLR1*, encoding LOX-1) as molecular markers of osteoclast activity and G-protein coupled receptors (GPCRs), complement C5a receptor 1 (*C5AR1*), somatostatin receptor 2 (*SSTR2*), and free fatty acid receptor 4 (*FFAR4*/GPR120), as novel molecular targets for modulating osteoclast differentiation and activity.

## Results

### Differentiating human osteoclasts

**Fig. 1:**
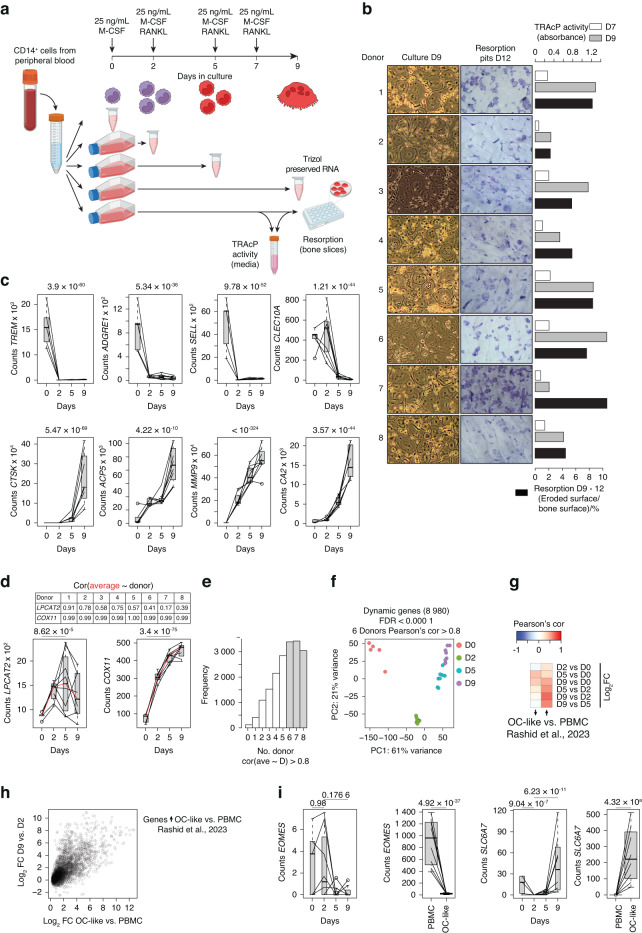
RNA collection during human osteoclast differentiation. **a** Schematic representation of the experimental setup. **b** Light microscopy images of mature osteoclasts on Day 9 and of resorption pits on Day 12, absorbance-based TRAcP activity in media at Days 7 and 9, and quantification of the percentage of eroded surface per bone surface resorbed by 50 000 mature osteoclasts from Days 9 to 12 for each of the eight donors. **c** Box plot of RNA-seq-based gene expression levels for monocyte (upper panel) and osteoclast-specific (lower panel) genes. **d** Box plot of RNA-seq-based gene expression levels for lysophosphatidylcholine acyltransferase 2 (*LPCAT2*) and cytochrome c oxidase copper chaperone (*COX11*). **e** Histogram reporting the frequency of genes grouped by the number of donors with a Pearson correlation greater than 0.8 to the average expression level. Genes within black bars were considered for further analysis. **f** Principal component analysis plot based on genes with differential (FDR < 0.000 1 between at least two timepoints) and reproducible (≥6 donors with Pearson’s correlation >0.8 to the average) expression during human osteoclast differentiation. **g** Heatmap showing the Pearson correlation for log twofold changes in gene expression during osteoclast differentiation according to Rashid et al.^20^ **h** Scatter plot comparing the log twofold changes between OC-like cells and PBMCs from Rashid et al.^20^ with log twofold changes occurring between Day 9 and Day 2 of osteoclast differentiation in the present study. Genes were selected based on upregulation (FDR < 0.01) within expression data from Rashid et al.^20^. **i** Box plot (band: mean; box: first and third quartiles; whiskers: 1.5 times the interquartile range) of RNA-seq-based gene expression levels for solute carrier family 6 member 7 (*SLC6A7*) in the present study (left panel) and from Rashid et al.^20^ (right panel)

To characterize transcriptional reprogramming during human osteoclastogenesis, we first determined the dynamic gene expression patterns of myeloid progenitors differentiating into mature osteoclasts. Therefore, human peripheral blood CD14^+^ monocytes from eight anonymous female blood donors aged 18–49 years were isolated and differentiated into osteoclasts using concomitant treatment with M-CSF for 9 days (Days 0-9) and RANKL for 7 days (Days 2–9). RNA was harvested on Day 0 and at Days 2, 5, and 9 after induction of differentiation (Fig. 1a). The activity of the osteoclast marker tartrate-resistant acid phosphatase (TRAcP) in the cell culture media was analyzed on Days 7 and 9. Furthermore, resorptive activity was determined by seeding and incubating osteoclasts on Day 9 in bovine bone slices for 72 h.

We found that cells from all donors differentiated into TRAcP-expressing multinucleated osteoclasts capable of resorbing bone as previously reported.^16,17^ TRAcP and resorptive activity varied between donors (Fig. 1b). RNA-seq analyses revealed time-dependent downregulation of monocyte-specific genes (e.g., *TREM1*, *SELL*, and *CLEC10A*)^18^ and monocyte-macrophage markers (e.g., *ADGRE1*)^19^ and a similar time-dependent increase in the expression of osteoclast-specific marker genes (e.g., *CTSK*, *ACP5, MMP9*, and *CA2*) from Day 0 to Day 9 (Fig. 1c). Analyses of the variance in gene expression at different time points revealed 10 849 genes that were differentially expressed; i.e., the level of gene expression changed between at least two time points across donors. For some of these genes, such as *COX11*, the donors exhibited a highly similar temporal expression pattern, whereas the expression of other genes, e.g., *LPCAT2*, exhibited substantial interdonor variation (Fig. 1d). To avoid donor-dependent gene expression patterns, we included only differentially expressed genes with high similarity across donors (Fig. 1e, f). Thus, we obtained 8 980 genes that were differentially expressed during osteoclast differentiation and formed the basis of subsequent analyses.

By comparing our dataset with recently published gene expression data from human myeloid progenitors and differentiated osteoclasts,^20^ we found a strong correlation between the datasets for upregulated genes (Fig. 1G), particularly when comparing Days 2 and 9 of our differentiation protocol, for which we also reached similar magnitudes of gene induction (Fig. 1H). This finding aligns well with the fact that the authors used mononucleated cells after 2 days of stimulation with M-CSF as a starting point, while we used CD14^+^ monocytes.^20^ Therefore, we did not identify nonmonocyte genes, such as the CD8^+^-T-cell related gene *EOMES* (Fig. 1i, left panel), and we did not observe early gene regulation upon M-CSF stimulation, as exemplified by SLC6A7, which was subsequently strongly upregulated in mature osteoclasts in both datasets (Fig. 1i, right panel).

### RNA-seq reveals temporal gene expression patterns with distinct cellular functions and implications for human bone biology

**Fig. 2:**
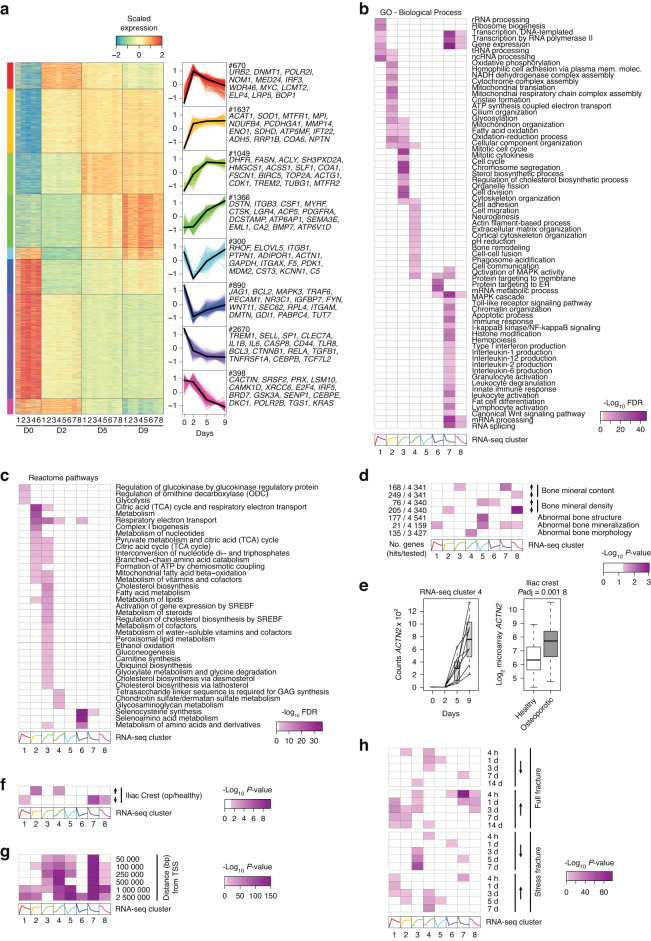
Temporal changes in gene expression patterns link osteoclast function to bone biology. **a** Heatmap showing scaled expression levels of the 8 446 differentially expressed genes among the eight k-means clusters for each sample. **b** Heatmap showing the false discovery rate (GOseq) for the enrichment of the gene clusters for biological process-annotated Gene Ontology (GO) terms. **c** Heatmap showing the false discovery rate (GOseq) for the enrichment of the gene clusters for pathways of the Reactome database. **d** Heatmap showing the *P* value (hypergeometric test) for the enrichment of the gene clusters for genes that increase or decrease bone mineral content or bone mineral density or genes causing abnormal bone structure, mineralization, and morphology in knockout mouse models from the International Mouse Phenotyping Consortium (IMPC). **e** Box plot of RNA-seq-based (with cluster membership, lines represent individual donors) gene expression levels for actinin alpha 2 (*ACTN2*) during human osteoclast differentiation and microarray-based (limma-based statistics) mRNA expression of *ACTN2* in iliac crest biopsies from healthy (*n* = 39) and osteoporotic (*n* = 27)^29^ subjects. **f** Heatmap showing the *P* value (hypergeometric test) for the enrichment of the gene clusters for genes up- or downregulated in iliac crest biopsies of osteoporotic patients (op) versus healthy controls.^29^ **g** Heatmap showing the enrichment of estimated bone mineral density (eBMD)-associated SNPs^33^ near genes whose expression changes dynamically during osteoclast differentiation. **h** Heatmap showing the *P* value (hypergeometric test) for the enrichment of the gene clusters for genes up- or downregulated during full or stress fracture in mice.^34^

Using k-means clustering, we observed that the 8 980 genes could be grouped into eight temporal gene expression patterns (clusters) (Fig. 2a). Among these, two clusters were characterized by a transient decrease or increase in gene expression levels, and six clusters were characterized by early (peak on Day 2), middle (peak on Day 5), or late (peak on Day 9) changes in gene expression levels. Gene Ontology (GO) and Reactome pathway analyses revealed cluster-specific enrichment of distinct biological processes (Fig. 2b, c), such as metabolic reprogramming and mitochondrial activation, which were linked to early upregulated genes (Cluster 2) (Fig. 2b). In line with this, we found distinct temporal profiles for metabolic genes involved in glucose metabolism and the tricarboxylic acid cycle (TCA)-mediated metabolism of fatty acids (Fig. 2c). The downregulated genes were barely linked to metabolic processes but important for cytokine production and immune cell activation. Canonical markers of osteoclast function, such as *CTSK*, *ACP5*, *DCSTAMP*, and *CA2* (ref.^16,21–24^), were among the late upregulated Cluster 4 genes that were involved in mature osteoclast-related processes, such as cytoskeleton organization, pH regulation, bone remodeling, cell migration, and cell‒cell fusion.^25–27^

To determine the associations of the differentially expressed genes with bone biology, we investigated whether there was an association between osteoclast gene expression profiles and genes associated with bone mineral density (BMD), bone mineral content (BMC), and/or bone morphology in knockout mouse models. Based on data from the International Mouse Phenotyping Consortium (IMPC),^28^ knockout phenotypes characterized by increased BMC and BMD (based on dual-energy X-ray absorptiometry [DXA]) were linked to genes repressed early during osteoclast differentiation, i.e., from monocytes to macrophages (Clusters 5, 6, and 7), while knockout phenotypes characterized by decreased BMC and BMD were linked to genes repressed during late osteoclast differentiation (Cluster 8) (Fig. 2d). Gene knockout phenotypes characterized by other skeletal defects on X-ray were linked to genes induced in mature osteoclasts (Cluster 4) and genes with decreased expression during osteoclastogenesis (Clusters 5-8). While disrupting osteoclast function is linked to bone phenotype-causing mutations in mice, these data indicate the existence of specific associations between subgroups of bone phenotypes and temporal gene expression patterns during osteoclast differentiation.

Next, we tested whether the differentially expressed gene patterns were aberrantly expressed in patients with osteoporosis. Using published microarray data on RNA in iliac crest bone tissue from 27 osteoporotic and 39 nonosteoporotic postmenopausal women,^29^ we found that the expression of *ACTN2*, a gene related to osteoclast fusion^30^ and a member of Cluster 4, was greater in bone from osteoporotic women (Fig. 2e). In a genome-wide context, we found an overlap between genes whose expression was upregulated in samples from osteoporotic women and genes whose expression was induced (Clusters 2 and 4) during osteoclast differentiation and vice versa for genes whose expression was repressed in both datasets (Clusters 7 and 8) (Fig. 2f). This finding suggested an increased abundance of osteoclast-specific markers and the absence of progenitor-related genes in bone in osteoporotic women. To further elucidate this phenomenon, we used published microarray data from peripheral blood monocytes (PBMs) from 73 nonosteoporotic pre- and postmenopausal Caucasian women grouped into low or high hip BMD groups assessed using DXA.^31,32^ However, in contrast to the clear enrichment patterns observed in the expression data from postmenopausal osteoporotic iliac crest biopsies, we could not find an association between genes whose expression was upregulated (Clusters 1–4) or downregulated (Clusters 5–8) during osteoclast differentiation and genes with altered expression in PBMs from nonosteoporotic pre- and postmenopausal women with low or high BMD (data not shown).

To test the association of differentiation-associated osteoclast genes with human genetics of bone mineral density, we used summary statistics from previously published GWAS data^33^ on heel quantitative ultrasound (eBMD) and tested the distribution of significant SNPs (*P* < 5 × 10^–8^) around the transcription start site of our clustered genes. We found that the density of SNPs associated with eBMD was greater for nearby genes with differential expression during osteoclast differentiation than for those with a genomic background (random distribution in the genome). This was especially the case for genes repressed after M-CSF stimulation (Cluster 7), late-induced genes (Cluster 4) and genes transiently repressed (Cluster 5) (Fig. 2g). These data indicate that genes linked to monocyte progenitors and mature osteoclasts, i.e., those whose expression strongly changes following differentiation from the macrophage stage, are likely to be affected by human sequence variations associated with alterations in eBMD.

Finally, we expanded our comparisons by moving from the steady-state analysis above to dynamic processes such as fracture healing. Using gene expression data^34^ from mice exposed to stress or a complete fracture, reflecting intramembranous and endochondral ossification processes, we observed a stronger and subsequently more coordinated association of the RNA-seq clusters with genes that are upregulated during the time course of fracture healing, especially in bones undergoing a full fracture (Fig. 2h). Taken together, these analyses show that distinct gene expression profiles throughout human osteoclastogenesis are implicated in bone development, bone remodeling during disease and fracture repair, as well as the genetics of bone density.

### Transcriptional networks drive osteoclast differentiation

**Fig. 3:**
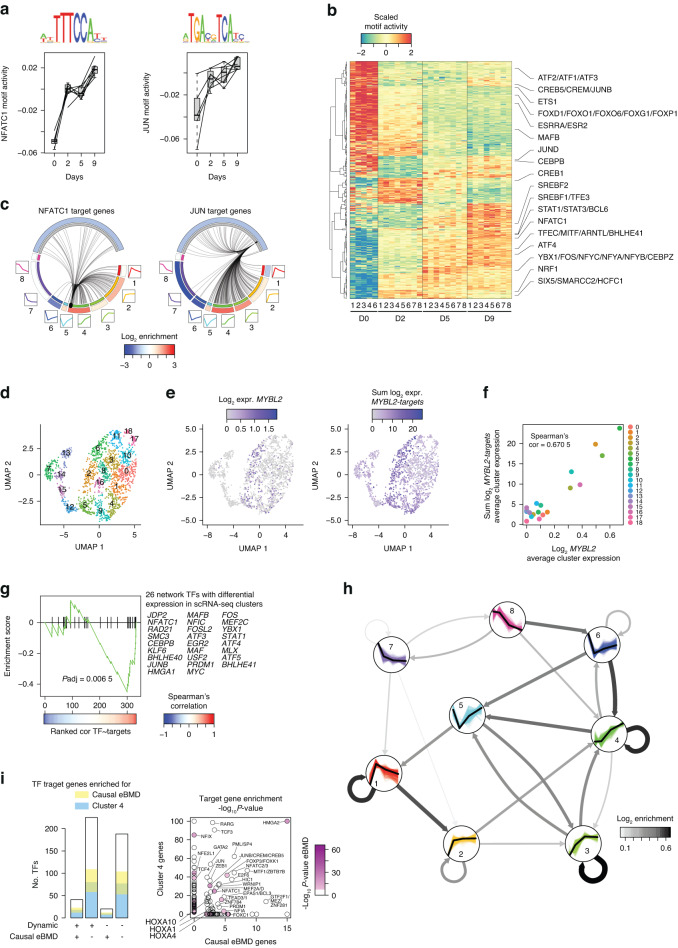
Machine learning highlights the transcriptional networks involved in human osteoclastogenesis. **a** Box plot of RNA-seq-based motif activity using ISMARA for NFATC1 and JUN during human osteoclast differentiation. Lines represent individual donors. **b** Heatmap showing the motif activity of transcription factors with differential activity (*P* value < 0.001) during human osteoclast differentiation. **c** Circular plot showing the ISMARA-predicted target genes of NFATC1 and JUN. **d** UMAP plot of scRNA-seq data from in vitro differentiated human osteoclasts on Day 14 of differentiation. **e** Gene expression levels of *MYBL2* and the sum of MYBL2 target genes in a UMAP plot of differentiated human osteoclasts at the single-cell level. **f** Average cluster expression levels of *MYBL2* versus the sum of MYBL2 targets in differentiated human osteoclasts at the single-cell level. **g** Gene set enrichment analysis of 26 transcription factors with cluster-specific expression patterns among the 329 transcription factors that were ranked according to Spearman’s correlation for transcription factor and target gene expression at the cluster level (as illustrated for *MYBL2* in 3 F). **h** Network enrichment analysis (NEAT) showing significantly enriched regulatory relationships within and between RNA-seq clusters based on the ISMARA-predicted target genes. **i** Genome-wide associations and their predicted causal genes for estimated bone mineral density (eBMD) were filtered for transcription factor information

We next investigated whether the temporal changes in gene expression were linked through regulatory networks, i.e., whether gene induction during the late stages of osteoclast differentiation was a consequence of early changes in gene expression. We applied the machine learning algorithm “Integrated System for Motif Activity Response Analysis” (ISMARA)^35^ to model transcription factor activity and to predict target genes of each transcription factor using gene expression data and motif occurrences in promoter regions. Importantly, the algorithm predicted a strong increase in the motif activity of two key transcription factors involved in osteoclast differentiation, NFATC1 and JUN^36^ (Fig. 3a). We also found that more than half of the 682 transcription factors that were found in the ISMARA motif database and our expression dataset exhibited changes in activity during differentiation (Fig. 3b); these genes included many of the transcription factors annotated in the GO terms “osteoclast differentiation” or “bone resorption”. In addition to the changes in activity, ISMARA predicts target genes for a given transcription factor, i.e., genes that are very likely to depend on the presence of that specific factor in the given expression dataset. In accordance with the continuous increase in *JUN* and *NFATC1* activity during osteoclast differentiation and their documented role in the maturation of osteoclasts,^36^ we found that most of the predicted target genes, i.e., members of Cluster 4, were upregulated during the late phase of differentiation (Fig. 3c). To test this model for target gene prediction, we performed coexpression analysis of transcription factors and their predicted target genes on published single-cell RNA-seq (scRNA-seq) data from in vitro differentiated human osteoclasts.^37^ Due to the sparse nature of the scRNA-seq data, we first performed deep clustering to estimate the correlation between the coexpression of transcription factors and predicted targets at the cluster level (Fig. 3d). As exemplified by the predicted target genes for MYB-related protein 2 (*MYBL2*), a transcription factor known to regulate cell cycle genes,^38^ which were enriched in Cluster 3 (data not shown), we found strong overlap between the expression of MYBL2 and the predicted target genes at both the single-cell (Fig. 3e) and cluster (Fig. 3f) levels. Importantly, similar associations were observed only among transcription factors with cluster-specific expression patterns (Fig. 3g), suggesting that the expression of target genes follows the expression pattern of the regulating transcription factors.

We next constructed a directed transcriptional network by combining the predicted target genes of all 682 transcription factors from the ISMARA database via network enrichment analysis.^39^ This analysis demonstrated that members of the early, transiently induced gene cluster (Cluster 1) were particularly important for the regulation of genes in the early and middle-induced clusters (Clusters 2 and 3), which in turn controlled the induction of osteoclast-specific genes (Cluster 4) (Fig. 3h). Using the transcriptional network approach, we showed that the proportions of transcription factors highly relevant for the regulation of mature osteoclast genes (Cluster 4) and/or causal eBMD genes^33^ were independent of the factor itself, which changed expression levels or was a causal eBMD gene (Fig. 3i). For example, we identified transcription factors, including members of the homeobox A and myocyte enhancer family, such as *HOXA10* and MEF2A, that are predicted to regulate genes related to mature osteoclasts and bone mineral density despite not being differentially expressed or associated with eBMD SNPs (Fig. 3I). Taken together, these network analyses demonstrated associations in the stepwise remodeling of the transcriptional networks regulating osteoclast differentiation, i.e., feedforward loops in gene regulation to overcome transitions between states of cellular differentiation in osteoclasts. In addition, our data suggested that network-based analysis can predict transcription factors important for osteoclast function based on the level of transcription factor activity, i.e., factors that contribute to the regulation of osteoclast genes through post-transcriptional mechanisms.

### Subgrouping of osteoclast transcriptional networks

**Fig. 4:**
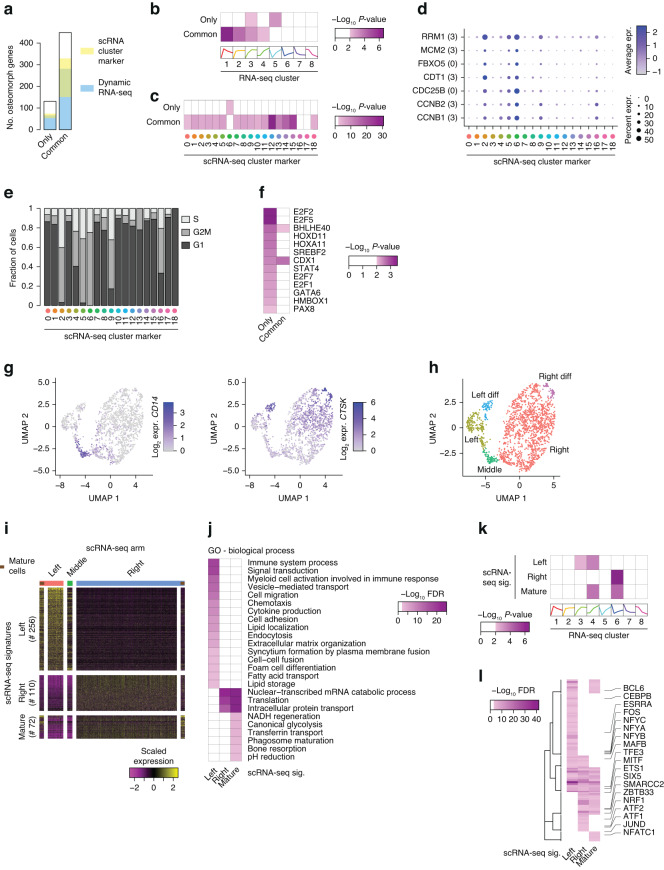
Subpopulation specificity of osteoclast transcriptional networks. **a** Bar plot showing overlap of osteomorph-selective (only) and osteomorph-osteoclast-selective (common) genes, with genes being differentially expressed throughout osteoclast differentiation at the bulk level (Fig. 2a – dynamic RNA-seq) and genes being cluster-specifically expressed in the scRNA-seq of mature osteoclasts (Fig. 3d – scRNA cluster marker). **b** Heatmap showing the *P* value (hypergeometric test) for the enrichment of the bulk RNA-seq gene clusters in Fig. 2a for the osteomorph-selective (only) and osteomorph-osteoclast-selective (common) genes. **c** Heatmap showing the *P* value (hypergeometric test) for the enrichment of the scRNA-seq cluster markers in Fig. 3d for the osteomorph-selective (only) and osteomorph-osteoclast-selective (common) genes. **d** Dot plot showing the cluster expression levels of the osteomorph-selective genes that overlap with the markers of scRNA-seq Cluster 6 in Fig. 3d. **e** Bar plot quantifying the cell cycle score according to the scRNA-seq data of mature osteoclasts. **f** Heatmap showing the unadjusted *P* values (hypergeometric test) for the enrichment of ISMARA-predicted transcription factor target genes for the osteomorph-selective (only) and osteomorph-osteoclast-selective (common) genes. **g** *CD14* and *CTSK* gene expression levels on a UMAP plot of differentiated human osteoclasts at the single-cell level. **h** UMAP plot of scRNA-seq data from in vitro differentiated human osteoclasts on Day 14 of differentiation clustered into two subpopulations of differentiating cells (left and right) and one population of undifferentiated cells (middle connective piece). **i** Heatmap of gene expression levels for left-, right- and mature-specific gene groups. **j** Heatmap showing the false discovery rate (GOseq) for the enrichment of the scRNA-seq signatures in 4I for biological process-annotated GO terms. **k** Heatmap showing the *P* value (hypergeometric test) for the enrichment of the bulk RNA-seq gene clusters in 2 A for the scRNA-seq signatures in 4I. **l** Heatmap showing the Benjamini–Hochberg adjusted *P* values (hypergeometric test) for the enrichment of ISMARA-predicted transcription factor target genes for the scRNA–seq signatures in 4I

As it was recently suggested that mature murine osteoclasts can undergo fission into osteomorphs, which are transcriptionally distinct from osteoclasts and macrophages and can be recycled back into osteoclasts,^40^ we questioned whether our gene expression data could be used to subgroup osteoclast transcriptional networks and provide insight into the transcriptional regulation of osteomorph-related genes. Using previously published gene signatures of osteomorphs, osteoclasts and monocytes in vivo,^40^ we found that the human orthologs of the 132 genes that were selectively expressed at high levels in osteomorphs (only) and of the 448 genes that were highly expressed in both osteomorphs and osteoclasts (commonly) were often dynamically expressed within our dataset and among the variable genes of the previously published human osteoclast single-cell RNA-seq data^37^ (Fig. 4a). Specifically, osteomorph-selective genes showed distinct enrichment from common osteomorph and osteoclast genes when aligned with our time course RNA-seq clusters (Fig. 4b) and marker genes of the single-cell RNA-seq clusters (Fig. 4c). Furthermore, osteomorph-selective genes were linked to cell proliferation, as highlighted by the strong overlap with RNA-seq Cluster 3 (Figs. 4b and 2b) and by the expression of osteomorph-selective genes in scRNA-seq clusters (Fig. 4d) with cells in proliferating cell cycle states (Fig. 4e). Using hypergeometric tests and our transcriptional network, we identified members of the *E2F* and *HOX* gene families that specifically regulate osteomorph-selective genes (Fig. 4f). However, this association was lost when correcting for multiple testing. Although neither our study nor that of Omata et al.^37^ provided culture conditions that support the presence of osteomorphs, we found that osteomorph-selective genes exhibit distinct dynamic expression patterns throughout human osteoclastogenesis, are partially linked to the cell cycle, and are likely regulated by transcription factors distinct from those regulating common osteoclast genes.

Expanding our analysis with previously published scRNA-seq data,^37^ we found that the scRNA-seq dataset contained two groups of mature osteoclasts at the top of two arms that emerged from monocytes (Fig. 4g, h). Therefore, we assessed the biological functional differences between the two arms and found that the left arm-selective genes were enriched among biological processes such as lipid metabolism, cytokine production and cell migration, whereas the right arm-selective genes were enriched among mitochondrial processes, ATP transport, and translation (Fig. 4i, j). Notably, the left- and right-arm selective genes also exhibited distinct temporal expression patterns in our bulk RNA-seq profiles (Fig. 4k) and, importantly, could be clustered separately according to our gene regulatory network (Fig. 4l). Here, the well-known osteoclast transcription factors JUN and NFATc1 were important for mature osteoclast genes independent of the subpopulation, while other osteoclast-associated transcription factors (GO terms ‘osteoclast differentiation’ and ‘bone resorption’) showed specificity for the genes of the left (e.g., CEBPB, FOS, BCL6) or right arm (e.g., ATF1, NRF1, SIX5). Although it is unknown whether these differences were caused by one arm containing immature and nonfused mature osteoclasts or the other containing mainly fully fused osteoclasts, these analyses demonstrated that subpopulation-selective osteoclast genes have distinct temporal expression patterns and that osteoclast subtype specificity is defined by the interaction of a core osteoclast transcriptional network (e.g., NFATc1) with subtype-specific transcription factors.

### Differentially expressed GPCRs during human osteoclast differentiation

From the perspective of identifying potential antiosteoporotic treatment targets, we speculated whether our dataset could be used to detect and test previously unrecognized regulators of osteoclast differentiation and activity. To test this possibility, we focused on the role of GPCRs in the transcriptional reprogramming of osteoclast differentiation, as GPCRs are implicated in bone biology and represent feasible therapeutic targets accounting for approximately 34% of FDA-approved drugs (2017) that target a total of 108 different GPCRs.^41^ An example of this is teriparatide, a bone anabolic antiosteoporotic drug that targets the parathyroid hormone (PTH) receptor on osteoblasts or their precursors.^42^ Furthermore, 36 GPCRs are known to be associated with altered BMD, bone morphology, and bone-related diseases such as arthritis in humans.^43^ For example, activating the calcitonin receptor, a bona fide osteoclast marker, reduces osteoclast activity.^44^

**Fig. 5:**
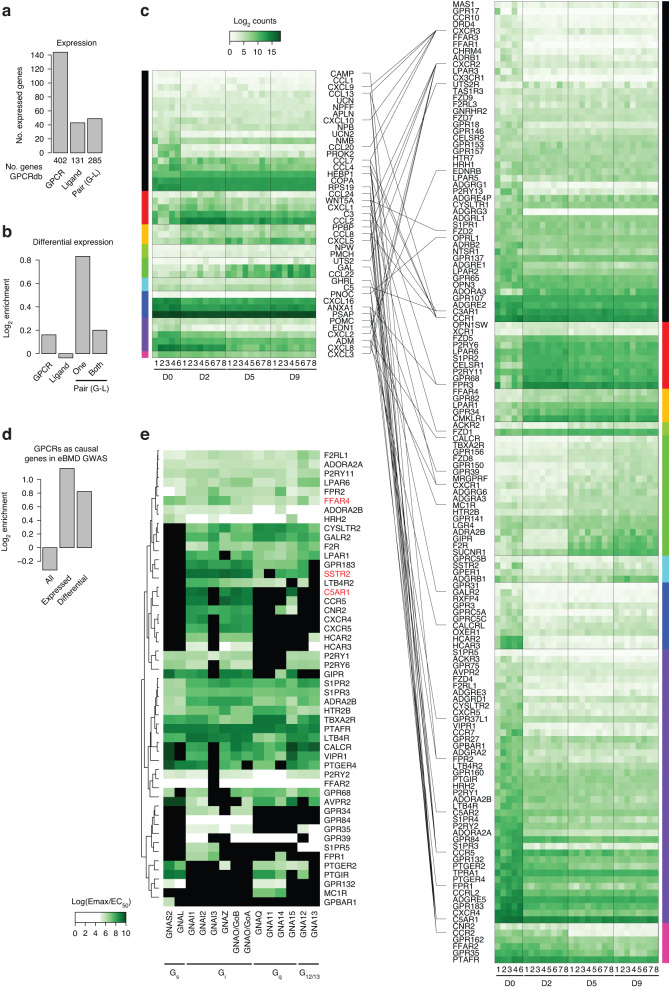
G-protein coupled receptors are highly differentially expressed during osteoclast differentiation. **a** Bar plot showing the frequency of nonolfactory G protein-coupled receptor (GPCR), gene-encoded GPCR ligand and receptor‒ligand (G-L) pairs from the GPCRdb expressed at the mRNA level during human osteoclast differentiation. **b** Bar plot quantifying the enrichment of GPCRs, gene-encoded ligands and pairs divided into one or both among the genes differentially expressed during human osteoclast differentiation. **c** Heatmap quantifying the gene expression levels of gene-encoded ligands (left panel) and GPCRs (right panel) during human osteoclast differentiation grouped according to gene clusters. **d** Bar plot quantifying the enrichment of nonolfactory GPCRs among the genes predicted to be causal genes in the eBMD GWAS, grouped into all and those expressed or differentially expressed during human osteoclast differentiation. **e** Heatmap quantifying the preferential coupling of GPCRs from RNA-seq Clusters 1–8 to G proteins according to the GPCRdb

Using the GPCR database (GPCRdb),^45^ we identified 144 GPCRs and 43 gene-encoded ligands expressed during osteoclast differentiation (Fig. 5a). Interestingly, to a minor extent, GPCRs but mostly ligand‒receptor pairs were enriched among the differentially expressed genes (Fig. 5b, c). Focusing on all human-encoded nonolfactory GPCRs, we did not find them to be enriched among the 835 genes predicted to be causally related to eBMD.^33^ However, this change occurred when GPCRs that were expressed or differentially expressed during osteoclast differentiation were grouped (Fig. 5d). Among the differentially expressed GPCRs, we found well-described regulators of osteoclast function, such as *CALCR* as well as *LGR4*, which encodes a leucine-rich repeat containing G-protein coupled receptor 4 that acts as a receptor for RANKL, thereby inhibiting osteoclast differentiation and activity;^46^ and *GIPR*, which encodes the receptor of the incretin hormone glucose-dependent insulinotropic polypeptide (GIPR), which inhibits human osteoclast differentiation and activity.^17^ Taken together, these data indicate a pivotal role for differentially expressed GPCRs and their ligands in the regulation of osteoclast differentiation and activity and, in turn, bone mineral density in humans.

Based on these findings, we hypothesized that other GPCRs could affect osteoclast differentiation or function. To test this hypothesis, we focused on differentially expressed GPCRs with known downstream G protein-coupling,^47^ which left us with 64 GPCRs (Fig. 5e). Of these, we selected three (marked in red in Fig. 5e), namely, *C5AR1* (complement 5a receptor 1), *SSTR2* (somatostatin receptor 2), and *FFAR4* (free fatty acid receptor 4/GPR120), because all these receptors are commercially available agonists and because their regulatory effects on human osteoclast differentiation and activity have not been previously investigated.

### C5AR1 activation impairs osteoclast differentiation due to its progenitor-restricted expression

**Fig. 6:**
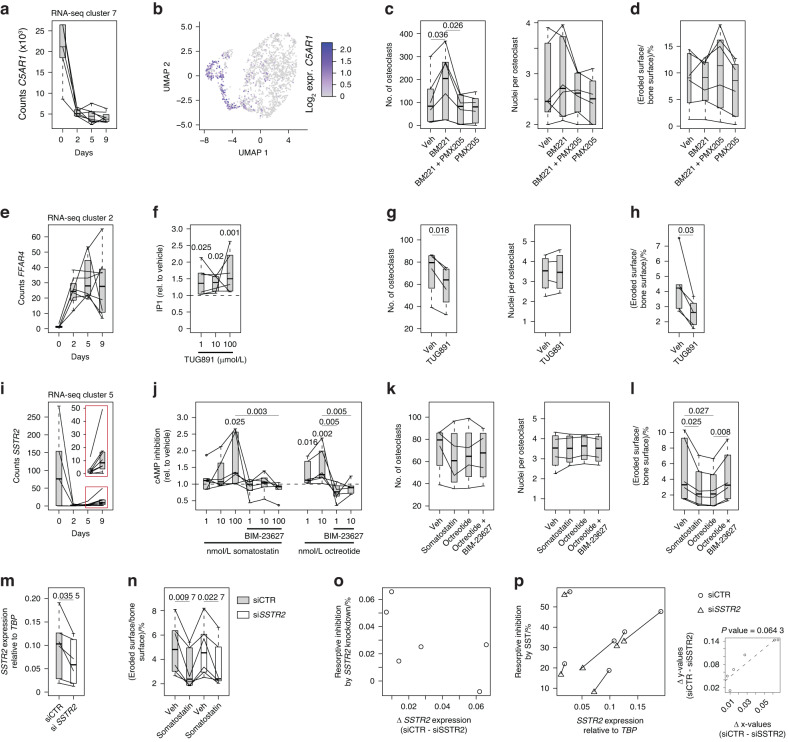
C5AR1, SSTR2 and GPR120/FFAR4 activation modulates osteoclastogenesis and/or resorptive activity of mature osteoclasts. **a** Box plot of RNA-seq-based gene expression levels of complement 5A receptor 1 (*C5AR1*). **b** UMAP plot of *C5AR1* gene expression in differentiated human osteoclasts at the single-cell level. **c** Box plot quantifying osteoclast numbers and nuclei per osteoclast after 9 days of osteoclast differentiation in the presence of the C5AR1 agonist BM221 (1 µg/mL) and/or antagonist PMX205 (5 µg/mL) (*n* = 6). **d** Box plot quantifying osteoclast resorptive activity as the percentage of eroded surface per bone surface for mature osteoclasts (Day 9) after 72 h on bovine bone slices in the presence of the C5AR1 agonist BM221 (1 µg/mL) and/or antagonist PMX205 (5 µg/mL) (*n* = 6). **e** Box plot of the RNA-seq-based gene expression levels of free fatty acid receptor 4 (*FFAR4*). **f** Box plot quantifying IP-1 levels in an HTRF assay, showing that the specific FFAR4 agonist TUG-891 increases IP-1 levels in mature osteoclasts (Day 9) after 30 min (*n* = 6). **g** Box plot quantifying osteoclast numbers and nuclei per osteoclast after 9 days of osteoclast differentiation in the presence of the FFAR4 agonist TUG-891 (10 nmol/L) (*n* = 4). **h** Box plot quantifying osteoclast resorptive activity as the percentage of eroded surface per bone surface for mature osteoclasts (Day 9) after 72 h on bovine bone slices in the presence of the FFAR4 agonist TUG-891 (10 nmol/L) (*n* = 5). **i** Box plot of RNA-seq-based gene expression levels of somatostatin receptor 2 (*SSTR2*). The inlet highlights the expression at Days 5 and 9. Lines represent individual donors. **j** Box plot quantifying the cAMP-Glo assay. **k** Box plot quantifying osteoclast numbers and nuclei per osteoclast after 9 days of osteoclast differentiation in the presence of the SSTR2 agonist somatostatin (100 nmol/L) and octreotide (10 nmol/L) and antagonism by BIM-23627 (100 nmol/L) (*n* = 4). **l** Box plot quantifying osteoclast resorptive activity as a percentage of eroded surface area per bone surface for mature osteoclasts (Day 9) after 72 h on bovine bone slices in the presence of the SSTR2 agonists somatostatin (100 nmol/L) and octreotide (10 nmol/L) and antagonism by BIM-23627 (100 nmol/L) (*n* = 6). **m** Box plot of real-time PCR-based gene expression levels of *SSTR2* relative to *TBP* in osteoclasts on Day 9 of differentiation (*n* = 6). **n** Box plot quantifying osteoclast resorptive activity after 72 h on bovine bone slices in the presence of the SSTR2 agonist somatostatin (100 nmol/L) as a percentage of the eroded surface area per bone surface (*n* = 6). **o** Scatter plot quantifying the percentage change in bone resorption following *SSTR2* knockdown (vehicle values from Fig. 8n: (siCTR – siSSTR2)/siCTR) relative to changes in *SSTR2* expression (values from Fig. 8m: siCTR – siSSTR2) (*n* = 6). **p** Scatter plot quantifying the percentage change in bone resorption in response to somatostatin (values from Fig. 8n: (Veh – somatostatin)/Veh) over relative *SSTR2* expression (values from Fig. 8m) (*n* = 6). The small scatterplot was used to quantify the delta of the x and y values of siCTR and si*SSTR2*, and the big scatterplot *P* value was calculated based on a linear regression model

The expression of *C5AR1*, a Cluster 7 member, was strongly decreased during osteoclast differentiation (Fig. 6a). *C5AR1* expression was detected via scRNA-seq in human monocytes and osteoclasts,^37^ in which C5AR1 expression was limited to undifferentiated cells (Fig. 6b), indicating that C5AR1 is not a functional receptor on mature osteoclasts. C5AR1 is a class A GPCR that predominantly couples to inhibitory G proteins (Gi) following ligand binding. This change leads to a decrease in intracellular cAMP levels^48^ and mediates innate immunity and promotes inflammation. Compared with wild-type mice, *C5ar1* knockout mice exhibit increased bone mass, reduced osteoclast formation and increased osteoblast numbers.^49^ In cellular models, the addition of supernatant from C5AR1-activated murine osteoblasts enhances murine osteoclastogenesis,^50^ and the activation of C5AR1 with C5a on human osteoblast-like cells induces interleukin-6 production, which may trigger bone resorption.^51^ Thus, C5a may couple osteoblast and osteoclast activity, and C5AR1 could have osteoclastogenic effects on human cells.

Treating differentiating osteoclasts and mature osteoclasts with the selective agonist BM221 (ref. ^52^) (1 µg/mL) showed that activation of C5AR1 enhanced osteoclastogenesis by increasing the number of newly formed osteoclasts (Fig. 6c). Importantly, these effects were reversed by the addition of PMX205 (5 µg/mL), a C5AR1 antagonist^53^ (Fig. 6c). BM221 did not affect the number of nuclei per osteoclast or the resorptive activity of mature osteoclasts (Fig. 6d), demonstrating that C5AR1 activation solely affects the quantity of osteoclasts.

### FFAR4 activation impaired osteoclast differentiation and decreased the resorptive activity of mature osteoclasts

The expression pattern of *FFAR4*, a member of Cluster 2, peaked on Day 2 and remained high throughout differentiation (Fig. 6e). Unlike that of *C5AR1*, the expression of *SSTR2* was not detected in the scRNA-seq data of human osteoclastogenesis. FFAR4 is a class A GPCR that, upon ligand binding, stimulates the generation of inositol 1,4,5-trisphosphate (IP3). This change induces intracellular calcium mobilization and phosphorylation of ERK via Gq/11-mediated signaling pathways.^54^ *Ffar4* expression in mice is strongly upregulated during osteoclastogenesis and negatively regulates osteoclast differentiation, function, and survival.^55^ Treating human CD14^+^ monocytes with docosahexaenoic acid (DHA), an n-3 fatty acid ligand for several free fatty acid receptors, including FFAR4, has been reported to inhibit osteoclast formation and activity and to downregulate the expression of genes important for osteoclast function, such as *DCSTAMP* and *NFATC1* (ref.^56^). However, as it remains unknown whether these inhibitory effects of DHA are mediated via FFAR4 activation, we further analyzed the effect of FFAR4 agonism on osteoclast differentiation and activity using the selective FFAR4 agonist TUG-891 (ref.^57^).

To determine the functionality of FFAR4 in human osteoclasts, we first measured the levels of IP1, a stable downstream metabolite of IP3, upon exposure to TUG-891 using an HTRF assay. We observed a significant increase in IP1 at all concentrations investigated (0.1, 1, and 10 μmol/L), with the most potent effect occurring at 10 μmol/L (Fig. 6f). We showed that FFAR4 activation impaired osteoclast differentiation by reducing the number of osteoclasts but not the number of nuclei per osteoclast (Fig. 6g). Finally, treating mature osteoclasts seeded on bone slices with TUG-891 inhibited resorptive activity (Fig. 6h), demonstrating that FFAR4 activation inhibits osteoclastogenesis by limiting the initial maturation of osteoclasts and the resorptive activity of mature human osteoclasts.

### SSTR2 activation did not change osteoclast differentiation but decreased osteoclast activity

The expression pattern of *SSTR2*, a member of Cluster 5, transiently decreased during osteoclast differentiation, with high expression occurring on Day 0 in some donors and upregulated during late differentiation in all donors (Fig. 6l). Like in the case of *FFAR4*, the expression of *SSTR2* was not detected in the scRNA-seq data of human osteoclastogenesis. SSTR2 is a class A GPCR that couples to inhibitory G proteins (Gi or Go) following ligand binding,^58^ leading to a decrease in intracellular cAMP levels. SSTR2 activity can be modulated by the somatostatin analog octreotide, which is used clinically to control the growth of and secretion of hormones from neuroendocrine tumors, such as insulinomas and growth hormone-secreting pituitary tumors.^59^ Furthermore, octreotide acutely increases the expression of markers of bone resorption and formation in healthy subjects.^60^

To determine the function of SSTR2 in human osteoclasts, we first measured cAMP signaling after exposure to either somatostatin or octreotide with or without pretreatment with the specific SSTR2 antagonist BIM-23627 (ref. ^61^) using cAMP-Glo assays in mature (Day 9) osteoclasts (Fig. 6j). We observed that somatostatin and octreotide dose-dependently decreased cAMP levels, although somatostatin had effects only at the highest concentration investigated (100 nmol/L). Importantly, the effects on cAMP were reversed when the cells were pretreated with BIM-23627. Treatment with somatostatin or octreotide did not affect osteoclast differentiation, as assessed by the total number of mature osteoclasts or the number of nuclei per cell in mature osteoclasts (Fig. 6k). However, both somatostatin (100 nmol/L) and octreotide (10 nmol/L) inhibited the resorptive activity of mature osteoclasts, and this effect was abolished when osteoclasts were pretreated with BIM-23627 (100 nmol/L) (Fig. 6l).

In contrast to C5AR1 and FFAR4, the function of SSTR2 in osteoclasts has not yet been studied in knockout animal studies. Therefore, we combined siRNA-mediated loss-of-function studies of human mature osteoclasts (Fig. 6m) with somatostatin treatment (Fig. 6n) to determine the specificity of pharmacological SSTR2 stimulation. siRNA-mediated decreases in the *SSTR2* mRNA level varied across donors, with a maximum 50% knockdown efficiency. Although donor-specific loss of *SSTR2* expression did not align with changes in basal resorptive activity (Fig. 6o), the SSTR2 knockdown efficiency clearly correlated with a decrease in the antiresorptive effect of somatostatin treatment (Fig. 6p). Taken together, these findings demonstrate that SSTR2 is a functional differentially expressed GPCR that, upon activation, has antiresorptive effects on mature human osteoclasts.

### Differential expression analysis for donor-specific resorption activity identifies resorption-associated markers in progenitor cells

**Fig. 7:**
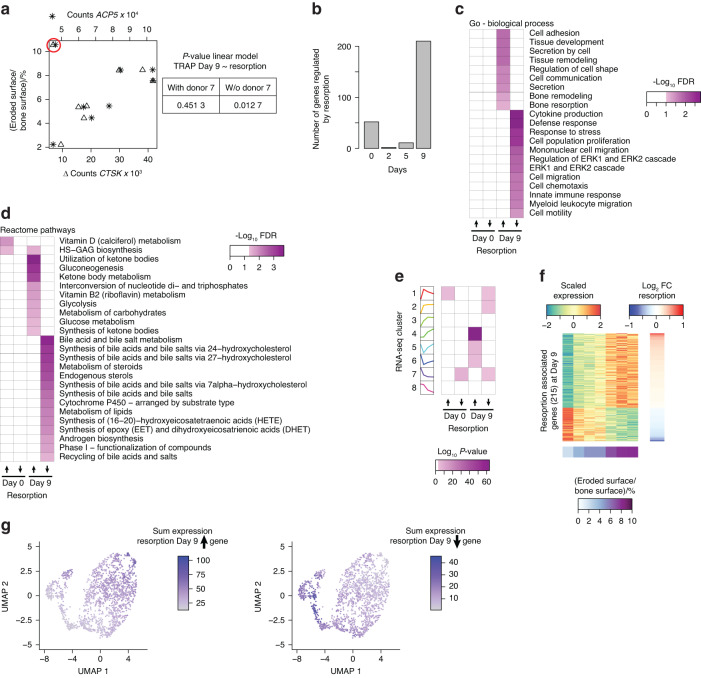
Gene expression in monocytes and differentiated osteoclasts aligns with cellular resorption activity. **a** Scatter plot of the resorptive effect of *CTSK* (lower x-axis) and *ACP5* (upper x-axis) on Day 9 of osteoclast differentiation. Donor 7 is highlighted with a red circle. The table shows the linear regression model for resorption and TRAP activity (Day 9) with and without donor 7. **b** Bar plot depicting the number of genes differentially expressed at Days 0, 2, 5, and 9 of osteoclast differentiation associated with resorptive activity (continuous DEseq2 model with FDR < 0.01). **c** Heatmap showing the false discovery rate (GOseq) for the enrichment of genes (Days 0 and 9) upregulated or downregulated with resorptive activity for biological process annotated Gene Ontology (GO) terms. **d** Heatmap showing the false discovery rate (GOseq) for the enrichment of genes (Days 0 and 9) upregulated or downregulated with resorptive activity for pathways of the Reactome. **e** Heatmap showing the p value (hypergeometric test) for the enrichment of the gene clusters for genes (Days 0 and 9) upregulated or downregulated with resorptive activity. **f** Heatmap showing scaled expression levels of the 215 genes related to resorptive activity on Day 9 of osteoclast differentiation that are differentially expressed. Row ranking based on log twofold change with resorption and column ranking based on resorptive activity of the donor cells. **g** UMAP plot showing the summed expression levels of resorption-associated genes whose expression was upregulated (left panel) or downregulated (right panel) on Day 9 of osteoclast differentiation

The donors used in this study were healthy women aged 18–49 years who were approved for blood donation. Nonetheless, we observed that the resorption activity was highly donor specific. Therefore, we questioned whether our expression data could reveal molecular signatures that are linked to the donor-specific resorption activity of cells. As expected, the expression of mature osteoclast genes that are linked to the differentiation status of cells, such as *CTSK* and *ACP5*, was strongly correlated with resorptive activity (Fig. 7a), except for that of donor 7, which was considered an outlier and was excluded from this analysis due to its high resorption capacity compared to its low TRAcP activity (Figs. 1b and 7a). Using a linear model in DESeq2 (ref.^62^), we did not find a single gene to be consistently associated with the resorptive activity of the mature osteoclasts at any time point, although many genes were regulated at Days 0 and 9 of differentiation (Fig. 7b). This finding suggested the presence of a gene signature within mature cells prior to differentiation, which is important for the resorptive activity of the cells. In contrast to the former, the latter gene signature did not show noteworthy enrichment for known biological pathways (Fig. 7c) or metabolic processes (Fig. 7d). Reasonably, mature osteoclast genes that increase in expression with resorption were enriched for osteoclast terms (Fig. 7c) and strongly overlapped with genes upregulated during late osteoclast differentiation (Fig. 7e). Focusing on mature osteoclast genes, we found 148 upregulated genes and 67 downregulated genes related to resorption (Fig. 7f). These two genes also exhibited high expression according to the scRNA-seq data^37^ of mature osteoclasts and undifferentiated precursors (Fig. 7g). Unfortunately, we could not distinguish whether the resorption-associated gene signature of mature osteoclasts was due to donor-specific expression patterns, interexperimental variation in differentiation efficiency, or both. Therefore, we decided to continue with the resorption-associated gene signature of CD14^+^ monocytes.

**Fig. 8:**
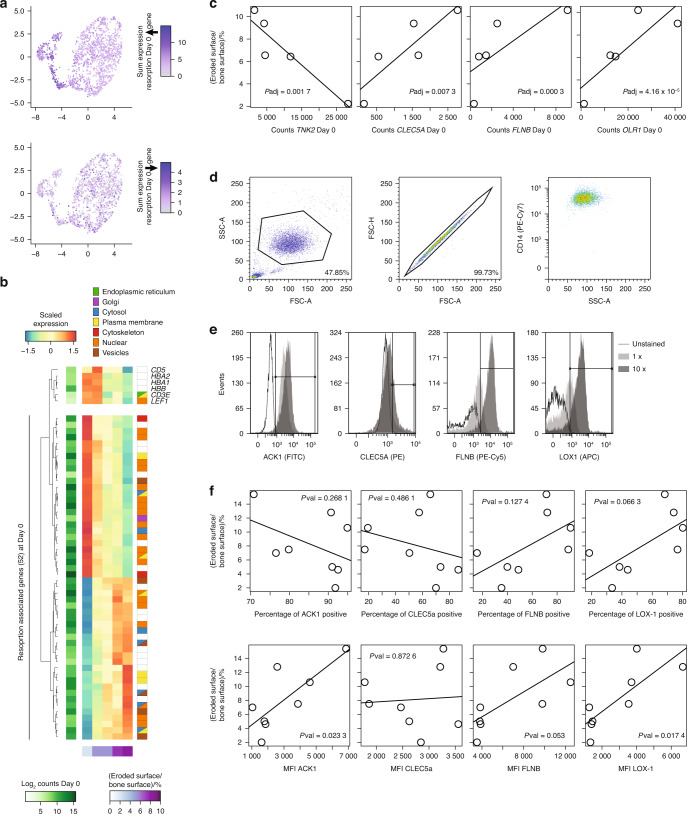
Monocytes represent surface protein markers that are predictive of resorption activity. **a** UMAP plot showing the summed expression levels of resorption-associated genes whose expression is upregulated (left panel) or downregulated (right panel) on Day 0 of osteoclast differentiation. **b** Heatmap showing scaled expression levels of the 52 resorption-associated genes in CD14^+^ monocytes (Day 0). **c** Scatter plot of the resorptive activity with the expression of tyrosine kinase nonreceptor 2 (*TNK2*, coding for ACK1), c-type lectin domain containing 5 A (*CLEC5A*), filamin B (*FLNB*), and oxidized low-density lipoprotein receptor 1 (*OLR1*, coding for LOX-1) on Day 0 of osteoclast differentiation. **d** Gating strategy for intact singlets of CD14^+^ monocytes. **e** Histograms illustrating the fluorescence intensity of CD14^+^ monocytes unstained or stained with antibodies against ACK1, CLEC5A, FLNB, or LOX-1 at two concentrations. **f** Percentage of positive cells (upper panel) and total fluorescence intensity (lower panel) of ACK1, CLEC5A, FLNB, and LOX-1 staining of CD14^+^ monocytes on Day 0 of osteoclast differentiation against the resorption activity of Day 9 differentiated osteoclasts that had been incubated on bone slices for 72 h (*n* = 8)

The resorption-associated gene expression signature of CD14^+^ monocytes could not be linked to any molecular function (Fig. 7c, d) and was highly distinct from the resorption-associated genes expressed in mature osteoclasts. Nevertheless, resorption-associated genes that are upregulated in CD14^+^ monocytes and those that are downregulated in mature osteoclasts showed similar overlaps with our RNA-seq clusters (Fig. 7f) and with previously published scRNA-seq data^37^ (Fig. 7g, right panel; Fig. 8a, upper panel). We next used protein localization information from the human protein atlas to identify surface molecules related to resorption-associated gene expression in CD14^+^ monocytes (Fig. 8b). We selected four gene candidates (Fig. 8c), namely, tyrosine kinase nonreceptor 2 (*TNK2*, encoding ACK1), C-type lectin domain containing 5 A (*CLEC5A*), filamin B (*FLNB*), and oxidized low-density lipoprotein receptor 1 (*OLR1*, encoding LOX-1). Except for CLEC5A, flow cytometry analysis of CD14^+^ monocytes (Fig. 8d) revealed a strong shift in the antibody-induced fluorescence intensity (Fig. 8e). For both FLNB and LOX-1 (*OLR1*), we observed an increase in the expression of cell surface proteins on CD14^+^ monocytes that gave rise to osteoclasts with high resorption activity (Fig. 8f, lower panel). This difference was also evident but less significant when the percentage of FLNB- or LOX-1-positive CD14^+^ monocytes was used (Fig. 8f, upper panel). Importantly, these associations were in line with the prediction from gene expression analysis (Fig. 8c), which was not the case for ACK1 (*TNK2*). Overall, we demonstrated that gene expression profiling of CD14^+^ monocytes can reveal surface molecules that can be used as molecular markers of osteoclast progenitors for the prediction of donor-specific resorptive activity.

## Discussion

With this study, we provide a genetic overview of transcriptional reprogramming during human osteoclast differentiation. We tested the validity of our findings through network analysis and through projection on GWASs and published expression datasets from subjects with and without osteoporosis. Network analyses support the stepwise remodeling of transcriptional networks, i.e., feedforward loops in gene regulation, to overcome transitions between states of cellular differentiation in osteoclasts. Furthermore, these analyses highlight subpopulation-selective regulatory circuits through projections on scRNA-seq datasets. Genes upregulated late during human osteoclastogenesis were strongly surrounded by SNPs associated with BMD and exhibited increased expression in iliac crest biopsies of osteoporotic patients, highlighting the importance of mature osteoclast genes in human bone mass maintenance and accelerated osteoclast maturation and activity in osteoporotic bone. Based on the distinct expression profiles of the three GPCRs, we highlight the potential of these datasets to identify molecular modulators of osteoclast differentiation, namely, C5AR1, SSTR2 and FFAR4/GPR120, that impact osteoclast differentiation and/or osteoclastic resorptive activity upon activation.

To our knowledge, these datasets offer the first overview of the transcriptional reprogramming that occurs during human osteoclast differentiation based on bulk RNA-seq data from CD14^+^ monocytes and data from another three timepoints during differentiation from eight human donors. Although several methods for generating human osteoclasts exist,^63^ we used a well-established method that includes magnetic isolation of CD14^+^ monocytes from the peripheral blood of females aged 18–49; these cells are differentiated into osteoclasts over 10 days using M-CSF from Days 0-9 and RANKL from Day 2 post-differentiation.^17,64^ Aligning our dataset with recently reported gene expression profiles of human osteoclasts,^20^ we found a strong resemblance for the induction of osteoclast-specific gene signatures and pathways but also refined distinctions that were expected due to differences in progenitor source and time point selection. Recently, the genetic trajectories of osteoclastogenesis in humans and mice were reported using scRNA-seq^37^ based on data from two time points, human and murine osteoclast differentiation. Since scRNA-seq data allow us to analyze cellular heterogeneity, we integrated scRNA-seq snapshots of differentiated human osteoclasts with our time course data. Here, we showed that molecularly distinct osteoclast subtypes differ in temporal transcriptional reprogramming and that the underlying transcriptional differences are based on the interaction of an essential core osteoclast network containing NFATc1 and subtype-specific transcription factors. Among the transcription factors that were highly important in our network analysis, we identified factors that selectively regulate mature osteoclast genes through post-transcriptional mechanisms. As such, we propose that HOXA10 and MEF2A are important transcriptional regulators of osteoclast maturity, but these factors are not regulated at the transcriptional level. HOXA10 is a well-known regulator of osteoblast differentiation that causes an increase in *Runx2* expression, which drives murine osteoblast maturation.^65^ In line with this, blocking HOXA10 represses osteoblastogenesis,^66^ and the deleterious effects of a high-fat diet on osteoblast differentiation correlate with decreased expression levels of HOXA10 in osteoblast precursors.^67^ Based on our network analyses, we propose that HOXA10 also plays a role in osteoclast differentiation. Although the potential role of HOX10 in osteoclast biology is currently unknown, consistent with the expression of mature osteoclast genes, *HOXA10* expression was increased in iliac crest biopsies from osteoporotic postmenopausal women compared to that in biopsies from nonosteoporotic women (data not shown).^29^ However, the genetic and clinical impact of changes in the expression levels of HOXA10 in humans need further investigation. Accordingly, we propose that MEF2A regulates osteoclast-specific and eBMD-specific genes. Although our data showed that *MEF2A* is not differentially expressed during human osteoclast differentiation, a previous study revealed differential *Mef2a* expression during murine osteoclast differentiation, with the highest expression occurring at the macrophage stage.^68^ Furthermore, *Mef2a* expression is needed for murine osteoclast development, and female mice lacking MEF2A exhibit an osteopetrotic phenotype.^68^ In addition, it has been proposed that MEF2A mediates the inhibition of *SOST*, which encodes sclerostin, by PTH in rat osteocytes,^69^ indicating a crucial regulatory role for MEF2A in several aspects of bone cell activity. However, the exact role of this gene in human osteoclast differentiation is unknown and needs further investigation.

While many of the temporal gene expression profiles were linked to specific molecular functions and metabolic pathways, genes in Cluster 5 could not be linked to any previously defined molecular or cellular function. According to our network enrichment analysis, Cluster 5 genes seemed to be an integral part of temporal transcriptional remodeling. In addition, Cluster 5 genes were highly enriched for bone-specific functions; they were closely related to eBMD SNPs, they were annotated as osteomorph-selective genes, and their expression was associated with resorption activity. Therefore, we suggest that these genes are strongly related to osteoclast function via pathways that have not yet been defined.

Based on our findings, we hypothesized that differentially expressed GPCRs can regulate osteoclast differentiation and activity. This effect was tested by pharmacological modulation of three differentially expressed GPCRs identified in our expression dataset. First, we tested the effects of C5AR1 activation on human osteoclast differentiation and activity. Importantly, there are two genes encoding the receptors for complement factor C5a, namely, *C5AR1* and *C5AR2*, which both presented decreasing gene expression patterns during osteoclast differentiation. To ensure specificity in activating C5AR1, we chose the selective C5AR1 agonist BM221 (ref. 52). In line with our findings, *C5ar1* knockout mice exhibit an osteopetrotic phenotype and a decreased number of osteoclasts.^49^ Whereas exposure of murine Raw264.7 cells to C5AR antagonists reduced the expression of markers of osteoclast differentiation,^70^ we did not observe a direct effect of the selective C5AR1 antagonist PMX205 on osteoclast differentiation. Although BM221 treatment increased the number of osteoclasts, we cannot account for the underlying mechanism, e.g., an increased number of osteoclast precursors capable of differentiating into mature osteoclasts. However, in an intestinal transplant rat model, BM221 treatment induced macrophage accumulation, which was blocked by PMX205 (ref.^71^), and a similar effect following C5AR1 activation during human osteoclast differentiation could explain the observed increase in osteoclast numbers. Interestingly, *C5AR1*, but not *C5AR2*, exhibited both precursor- and subtype-specific expression patterns according to the scRNA-seq data of human osteoclasts, which suggested that the increase in osteoclast numbers might be due to the increase in a specific subpopulation of cells. We speculate that the increased number of newly formed osteoclasts observed following C5AR1 activation could be explained by an increased number of so-called fusion founders, which are rare and small subsets of osteoclast precursors considered to initiate cell fusion.^72^ Whether this is the case and whether mature osteoclasts differ in their activity depending on whether they are descendants of *C5AR1*-expressing subpopulations will be of interest for future investigations.

Next, the effects of SSTR2 activation were assessed using somatostatin and octreotide. Although octreotide targets several somatostatin receptors, including SSTR2 and SSTR5, with high affinity and SSTR3 with low affinity, but not SSTR1 or SSTR4 (ref.^73^), our data revealed that *SSTR5* is not expressed at any time point during osteoclastogenesis and that *SSTR3* expression is very low after the monocyte stage. Furthermore, pretreatment with the specific SSTR2 antagonist BIM-23627 (ref. ^61,74^) reversed both the antiresorptive effects of octreotide and changes in cAMP, and siRNA-mediated downregulation of SSTR2 suppressed somatostatin-mediated antiresorptive activity, suggesting that the antiresorptive effects of treating osteoclasts with octreotide reflect the activation of SSTR2.

Free fatty acids are generally suitable GPCR ligands,^75^ and modulating GPCRs activated by free fatty acids with different pharmacological compounds has been the focus of several clinical trials as novel treatments for type 2 diabetes.^76^ Interestingly, FFAR4 activation suppresses food intake, enhances peripheral insulin sensitivity, and promotes insulin secretion, and FFAR4 has been proposed as a potential target in the future treatment of type 2 diabetes.^77^ In a recent study, coactivation of FFAR4 and two other free fatty acid receptors enhanced glucagon-like peptide 1 (GLP-1) secretion from the gut and reduced appetite in overweight individuals,^78^ which indicated that in addition to type 2 diabetes, obesity may also be a treatment target for FFAR4 activation. Here, we showed that activating FFAR4 inhibited the early maturation of osteoclasts by reducing the number of newly formed osteoclasts, which was opposite to the effects observed following C5AR1 activation and osteoclastic resorptive activity. As FFAR4 stimulation did not affect the number of nuclei per osteoclast, it may seem that FFAR4 activation does not influence osteoclast fusion. However, whereas we speculate that C5AR1 activation may increase the number of fusion founders, FFAR4 activation may reduce the number of fusion founders but not the number of fusion followers, the latter being nonstimulated osteoclast progenitors that are unable to initiate cell fusion alone.^72^ This finding is in line with preclinical studies in mice in which treatment with DHA inhibited inflammation-induced osteoclast formation via interaction with FFAR4 (ref.^79^), and the ability of fat-1 transgenic mice to resist ovariectomy-induced bone loss depended on the expression of *Ffar4* (ref.^80^). Taken together, these findings suggest that the clinical effects of FFAR4 treatment might be used not only for disorders such as type 2 diabetes and obesity but also the inhibition of osteoclast differentiation and activity.

The effects of pharmacological activation of SSTR2 and FFAR4 highlight that activation of differentially expressed GPCRs, independent of the gene expression profile, changes cell activity in mature osteoclasts. In line with these findings, we recently showed that activation of GIPR, a GPCR with a different gene expression profile than that of *SSTR2* and *FFAR4*, downregulated genes important for osteoclast function and inhibited both osteoclast differentiation and osteoclastic resorptive activity.^17^ While GIPR was highly expressed in mature osteoclasts and contained in Cluster 4, receptors for other key gastrointestinal hormones, such as the GLP-1 receptor and glucagon receptor, were not (data not shown). Interestingly, the in vitro antiresorptive effects of SSTR2 activation were similar to those observed after agonizing GIPR although the genes encoding the receptors were contained in different clusters and expressed at different levels in mature osteoclasts.^17^ This finding highlights that detailed temporal gene expression studies can identify stage- and subpopulation-specific expression patterns of GPCRs but that their regulatory effects on differentiation and resorption require detailed cell differentiation, activity, and signaling studies.

In addition to the transcriptional network driving osteoclast differentiation, we identified gene signatures in CD14^+^ monocytes and mature osteoclasts that are linked to the resorption activity of these cells. The resorption-associated gene program of mature cells can be biased by the contribution of a “more” or “less” efficient differentiation experiment. To estimate this confounder, multiple rounds of differentiation from the same donor need to be performed. In contrast, this experimental bias should be limited at the progenitor level, and subsequently, we verified that the protein expression of FLNB and LOX-1 was positively associated with the resorption activity of differentiated cells in vitro. FLNB is a key component of the cytoskeleton and has not been functionally linked to osteoclast biology; however, sequence variations as well as mutations in human *FLNB* are linked to bone structure^81^ and human skeletal disorders.^82^ One of the latter is spondylocarpotarsal syndrome, characterized by vertebral fusions, a phenotype that is recapitulated in *Flnb* knockout mice.^83,84^ LOX-1 is a receptor for oxidized low-density lipoproteins, and its deficiency in *Olr1*^*-/-*^ mice has been shown to favor osteoclast fusion accompanied by decreased bone mass at steady state, while it also confers resistance to inflammation-induced bone loss.^85^ However, in human osteoclasts, *OLR1* upregulation has been found in activin A-induced giant osteoclasts.^86^ Clearly, LOX-1 levels are linked to osteoclast fusion; however, the effect on osteoclast function is highly context dependent.

Some limitations apply to this study. This study was based on the donation of monocytes from young healthy women. Therefore, potentially relevant information, including lifestyle factor, clinical information, and other genetic data, was unavailable. Another limitation is the use of female, presumably premenopausal, donors. Therefore, the reported findings may be different between male and postmenopausal donors. Due to filtering out the differentiation-associated genes for high donor-to-donor variation, we speculate that resorption-associated genes might be much more influenced by being limited to female donors only.

In conclusion, this study provides a genetic overview of the transcriptional reprogramming that occurs during osteoclast differentiation in premenopausal women and the implications of genes related to osteoclast differentiation for the etiology and genetics of human osteoporosis. This study identified molecular targets and predictive markers relevant for future studies on the regulation of osteoclast differentiation and activity to identify potential antiosteoporotic treatment targets.

## Materials and methods

### Compounds

The following compounds were used at the following concentrations according to the literature and purchased from the following companies: BM221 (ref. ^52^) (0.2 µg/mL, 1 µg/mL and 10 µg/mL; Product No. GLXC-24851; Glixx Laboratories, Inc., MA, USA); PMX205 (ref. ^87^) (5 µg/mL and 15 µg/mL; Product No. 5196; Bio-Techne Tocris, MN, USA); somatostatin-14 (ref. ^88^) (1, 10 and 100 nmol/L; Product No. S9129; Merck, London, UK); octreotide^88^ (1 and 10 nmol/L; Product No. O1014; Merck, London, UK); BIM-23627 (ref. ^74^) (1, 10 and 100 nmol/L; Product No. 4041642; Bachem, Bubendorf, CH); and TUG-891 (ref. ^54,57^) (0.1, 1 and 10 μmol/L; Product No. SML1914; Merck, London, UK). The compounds were diluted in dimethyl sulfoxide (DMSO).

### Generation of human osteoclasts

For RNA-seq, primary human osteoclasts were differentiated from human CD14^+^ monocytes isolated from buffy coats obtained from eight anonymous female blood donors aged 18–49 years. Monocytes were isolated as previously described.^89^ Following isolation, monocytes were seeded in five different T25 cell culture flasks (1.67 × 10^6^ cells/flask) (four used for RNA-seq and one used for bone resorption assays) in α-minimal essential medium (Gibco^TM^ MEM α, Thermo Fischer Scientific, Roskilde, DK) supplemented with 10% fetal bovine serum (Gibco^TM^ FBS, Thermo Fischer Scientific, MA, USA) and 1% penicillin/streptomycin (Thermo Fischer Scientific, MA, USA) and stimulated with 25 ng/mL M-CSF (Bio-Techne, Abingdon, UK). After two days, the medium was replaced with fresh medium containing M-CSF and RANKL (25 ng/mL each; Bio-Techne, Abingdon, UK); this process was repeated every 2-3 days thereafter fora total of seven days of stimulation with RANKL. The cells were maintained at 37 ^o^C and 5% CO_2,_ and microscopic validation of the presence of multinucleated cells was performed before the cells were used for the experiments.

For studies on C5AR1, SSTR2 and FFAR4, CD14^+^ monocytes were isolated from another 14 anonymous female blood donors aged 18-65 years; cells from three donors were used for analyses of both SSTR2 and FFAR4 (*n* = 6 for C5AR1, *n* = 6 for SSTR2, *n* = 5 for FFAR4), as previously described.^89^ Monocytes were seeded in T75 cell culture flasks (5 × 10^6^ cells/flask) and differentiated as described above. Sample collection was conducted in accordance with the principles of the Declaration of Helsinki, and informed consent was obtained from all the donors. Approval for collection of samples in the UK was obtained from local ethics committees (ERN_14-0446).

### RNA-seq

Cells in T25 cell culture flasks were lysed using 1 200 µL of TRIzol/mercaptoethanol (100:1) at Days 0, 2, 5, and 9. The cells were differentiated and seeded as described above with the following exemptions on Days 0 and 2. Four hours after incubation on Day 0, the αMEM with M-CSF was removed from one T25 flask, which was then washed in 5 mL of phosphate buffer solution (PBS) before the cells were lysed. For cells lysed on Day 2, M-CSF and RANKL (25 ng/mL each) were added to the T25 flasks and incubated for four hours. After four hours, the media were removed, and the T25 flasks were washed in 5 mL of PBS before the cells were lysed. On Days 5 and 9, the αMEM was removed from the T25 flask corresponding to one of these timepoints, and the cells were washed in 5 mL of PBS before they were lysed as described. Following lysis, the cells were stored at −80 °C and thawed once for RNA purification. RNA was purified using Econo Spin columns (Epoch Life Sciences, TX, USA), and the cells were stored at −80 °C before the final analyses. RNA sequencing was subsequently performed according to the manufacturer’s instructions (TruSeq 2, Illumina, CA, USA) using 2 µg of RNA for preparation of cDNA libraries. Sequencing reads were mapped to the human genome (GRCh38) using Spliced Transcripts Alignment to a Reference (STAR) software,^90^ and tag counts were summarized at the gene level using hypergeometric optimization of motif enrichment (HOMER).^91^ Differential gene expression was analyzed using DESeq2 (ref. ^62^) with a factor design (design = ~Timepoint + Donor), which regresses donor variation to determine differentiation-associated genes, and a linear variable (design: ~Resorption) was used for each individual time point to determine resorption-associated genes. Differentially expressed genes were selected based on an adjusted *P* value < 10^−4^ for differentiation-associated genes and an adjusted *P* value < 10^−2^ for resorption-associated genes. Gene Ontology (GO) and Reactome pathway^92^ analyses were performed using GOseqseq.^93^

### Motif activity and network analysis

Motif activity and target prediction were performed using the ISMARA algorithm^35^ by submitting bam files of the raw sequencing data. For network construction, target genes were selected based on a score above 2. The overlap of target genes with RNA-seq clusters was determined using a hypergeometric test. Network enrichment analysis was performed using NEAT.^39^ Regulatory transcription factors were determined using hypergeometric testing of the target genes for each transcription factor against the gene groups of interest followed by correction for multiple testing using the Benjamini–Hochberg procedure.

### Enrichment analysis of eBMD GWASs, published expression datasets, mouse knockout phenotypes, and osteomorph genes

GWAS summary statistics were retrieved from http://www.gefos.org/?q=content/data-release-2018 (ref.^33^). The eBMD SNPs were grouped into significant (*P* < 5 × 10^−8^) and nonsignificant (*P* > 5 × 10^−8^) groups and linked to genes using a variable window from 50 to 2 500 kb from the transcription start site. Enrichment significance was calculated by Pearson’s chi-square test, which compares the ratio of significant to nonsignificant SNPs for each cluster against the whole genome. Raw expression data for peripheral blood monocytes from subjects with low and high BMDs^31,32^ and iliacrest biopsies from healthy individuals and osteoporotic individuals^29^ were downloaded from the GEO GSE56815 and ArrayExpress E-MEXP-1618 cohorts. Differential expression analysis was performed with limma.^94^ Processed gene expression data for mouse fracture models^34^ and human osteoclasts in vitro^20^ were downloaded from the GEO datasets GSE152677 and GSE225974. Human orthologs from published mouse osteomorph genes (downloaded from Table S1 (ref. ^40^)) were grouped as described for osteomorph-selective and osteomorph-osteoclast-common genes. Data on mouse knockout phenotypes were collected from the International Mouse Phenotyping Consortia (http://ftp.ebi.ac.uk/pub/databases/impc/all-data-releases/latest/results/). Enrichment analysis to compare the overlap of gene groups was performed using either a hypergeometric test or gene set enrichment analysis.^95^

### scRNA-seq

The Cell Ranger count was generated by Yasunori Omata and Mario M. Zaiss.^37^ The count matrix was filtered for high-quality droplets using valiDrops^96^ and subsequently processed with Seurat^97^ for UMAP projection, cell cycle scoring, cluster definition, and identification of cluster-selective gene signatures. The expression sum of a gene group was calculated by extracting expression levels at the cell or cluster level and adding the expression values of the genes of interest. For the UMAP projection, the summed expression at the cell level was integrated back to the Seurat object. Processed Seurat objects are available at the open science framework: https://osf.io/9xys4/.

### Osteoclast resorption assays

For donors used for RNA-seq, on Day 9, multinucleated osteoclasts were removed from cell culture flasks by Accutase treatment. Briefly, the media was removed, and the cells were washed twice with PBS, followed by incubation with Accutase (Merck Life Science) at 37 °C for 5-8 min. The cells were then carefully removed using a cell scraper. The cells were centrifuged for 5 min at 1 500 r/min and resuspended in αMEM supplemented with 10% FBS. Ten microliters of cell suspension was mixed with 10 μL of trypan blue to assess cell viability and cell number. Thereafter, the cells were seeded on bovine cortical bone slices (0.4 mm thick) (BoneSlices.com, Jelling, DK) in 96-well plates at a density of 50 000 cells per bone slice in αMEM supplemented with 10% FBS, 25 ng/mL M-CSF and 25 ng/mL RANKL (*n* = 6 technical replicates per donor) and incubated for 72 h. After 72 h, the media were collected for subsequent TRAcP analyses, and the experiment was terminated by adding 200 μL of demineralized water to each bone slice. Bone slices were scraped with a cotton swab and stained with toluidine blue solution (1% toluidine blue, 1% sodium borate) (Merck Life Science, Søborg, DK) to visualize resorption excavations using a 100-point counting grid, and counting was performed using a 10 x objective of a BX53 Olympus microscope (Olympus, Tokyo, Japan). Bone resorption was assessed by calculating the total percentage of eroded surface material, as previously described.^98^

For the donors used for C5AR1, SSTR2 and FFAR4 activation, on Day 9, multinucleated osteoclasts were seeded on bovine cortical bone slices (0.4 mm thick) (BoneSlices.com, Jelling, Denmark) in 96-well plates at a density of 50 000 cells per bone slice in αMEM supplemented with 10% FBS, 25 ng/mL M-CSF and 25 ng/mL RANKL (*n* = 4-6 technical replicates per donor). The cells were allowed to settle for 40 min, after which vehicle, BM221 (1 µg/mL), or PMX205 (5 µg/mL) for the C5AR1 studies, somatostatin-14 (100 nmol/L) or octreotide (10 nmol/L) for the SSTR2 studies or TUG-891 (10 μmol/L) for the FFAR4 studies was added to the media. In studies involving combined agonist/antagonist exposure, cells were preincubated with 5 µg/mL PMX205 for 10 min before 1 µg/ml BM221 was added for C5AR1 studies or with 100 nmol/L BIM-23627 for 10 min before 10 nmol/L octreotide was added for SSTR2 studies. The cells were then incubated for 72 h. After 72 h, the experiments were terminated by adding 200 µL of demineralized water to each bone slice. Bone slices were scraped with a cotton swab and stained with toluidine blue solution (1% toluidine blue, 1% sodium borate) (Sigma‒Aldrich) to visualize resorption excavations as described above. Bone resorption was assessed by a blinded method by quantifying the total percentage of eroded surface area via light microscopy and a counting grid, as previously described.^98^ Statistical analyses were performed by paired t tests using GraphPad Prism.

### Quantification of the number of nuclei per osteoclast

CD14^+^ monocytes were isolated and seeded as described above. After 2 days, the cells were loosened with Accutase and seeded in 96-well plates (2.5 × 10^4^ cells per well). The cells were incubated with media containing 25 ng/mL M-CSF, 25 ng/mL RANKL and either vehicle (DMSO), 1 µg/mL BM221, 5 µg/mL PMX205, 5 µg/mL PMX205 + 1 µg/mL BM221, 100 nmol/L somatostatin-14, 10 nmol/L octreotide, 100 nmol/L BIM-23627 + 10 nmol/L octreotide or 10 μmol/L TUG-891 (*n* = 4 technical replicates, 4 donors). Media containing either vehicle or compounds as described above were refreshed after 3 and 5 days. After a total of 7 days of culture, the media was removed, and the wells were washed twice with PBS. The cells were then fixed with 3.7% formalin and methanol and stained with Giemsa and May-Grünwald (Merck, Watford, UK), as previously described.^99^ The number of multinucleated osteoclasts and their nuclei were counted systematically in every second counting field using a 10 x objective Axiovert 200 microscope (Zeiss, Oberkuchen, Germany) as previously described.^99^ Statistical analyses were performed by paired t tests using GraphPad Prism.

### Tartrate-resistant acid phosphatase 5b (TRAcP) activity analysis

TRAcP activity was measured in conditioned media from each of the bone resorption experiments from donors used for RNA-seq analyses. Conditioned medium (10 µL per well) was transferred in duplicate into a 96-well plate with 90 µL of TRAcP solution buffer (1 mol/L acetate, 0.5% Triton X-100, 1 mol/L NaCl, 10 mmol/L EDTA (pH 5.5), 50 mmol/L L-ascorbic acid, 0.2 mol/L disodium tartrate, 82 mmol/L 4-nitrophenylphosphate, all reagents from Sigma) and incubated at 37 °C in the dark for 30 min. The reaction was stopped by adding 100 μL of stop buffer (0.3 mol/L NaOH), and TRAcP activity was measured at an absorbance of 400 nm and 645 nm on a Synergy HT microplate reader (Biotek Instruments, VT, USA). Statistical analyses were performed by paired t tests using GraphPad Prism.

### cAMP-Glo assays

Mature osteoclasts were plated in 96-well white plates at a density of 6 250 cells per well and left to settle. At least four hours later, cAMP-Glo assays were performed following the manufacturer’s instructions (Promega, Chilworth, UK). The cells were exposed to either vehicle (DMSO) or the test compounds as described above in complete induction buffer (Promega) supplemented with 500 μmol/L 3-isobutyl-1-methylxanthine (IBMX) (Merck, London, UK) or 10 μmol/L forskolin (Tocris, Bristol, UK) for 30 min. Three technical replicates were performed for each of the *n* = 6 biological replicates. Luminescence was measured on a Pherastar FS (BMG Labtech, Aylesbury, UK). Responses were normalized to the vehicle control. Statistical analyses were performed using one-way ANOVA with the Kruskal‒Wallis test.

### IP1 assays

Mature osteoclasts were plated in 96-well white plates at a density of 6 250 cells per well and left to settle. At least four hours later, IP-one assays (Cisbio, Codolet, France) were performed according to the manufacturer’s guidelines. Three technical replicates were performed for each of the *n* = 6 biological replicates. The cells were exposed to the test compounds as described above and diluted in stimulation buffer (Cisbio, Codolet, France) for 30 min, followed by lysis in the supplied lysis buffer. The HTRF signal (at 665 nm and 620 nm) was read on a Pherastar FS (BMG Labtech, Aylesbury, UK) one hour later. Responses were normalized to the vehicle control. Statistical analyses were performed using one-way ANOVA with the Kruskal‒Wallis test.

### Transfection of mature osteoclasts with siRNA

Knockdown was essentially performed according to a previously published procedure.^100^ siRNA sequences (Horizon Discovery Biosciences, Cambridge UK) targeting *SSTR2* or a nontargeting control (ON-TARGETplus Nontargeting Control siRNA) dissolved in transfection buffer (Tebu-Bio, Roskilde, DK) were used to transfect human osteoclasts on Day 8 of differentiation using the GenMute^TM^ siRNA transfection reagent (Tebu-Bio, Roskilde, DK). After 24 h, the knockdown efficiency of SSTR2 was assessed via qPCR, or osteoclasts were loosened and seeded on bovine bone slices with or without 100 nmol/L somatostatin-14 to determine resorptive activity as described above.

### Real-time PCR

For quantification of the extent of *SSTR2* knockdown, RNA was extracted from osteoclasts transfected with either *SSTR2* siRNA or a nontargeting control agent using a TRIzol Plus RNA Purification Kit (Invitrogen) according to the manufacturer’s instructions. The concentration and quality of total RNA were measured using a NanoDrop 2000 (Thermo Scientific), and 500 ng of total RNA was reverse transcribed using a high-capacity cDNA reverse transcription kit (Applied Biosystems). The expression levels of *SSTR2* (Taqman probes: Thermo Fisher: #4331181) were quantified on a Viia 7 Real-time PCR device (Applied Biosystems) and normalized to those of *TBP* (for: GCC CGA AAC GCC GAA TAT; Rev: CCT CAT GAT TAC CGC AGC AAA) using Fast SYBR Green Master Mix.

### Flow cytometry

CD14^+^ monocytes were isolated, seeded, differentiated, and prepared for resorption assays as described above. In addition, CD14^+^ monocytes (2.5 × 10^6^ cells/mL) were resuspended in flow buffer (HBSS; Thermo Fisher, Glascow, UK) supplemented with BSA (0.5%, Sigma Aldrich, Damstad, D) and EDTA (2 mmol/L, Sigma Aldrich, St. Louis, MO, USA) and blocked with 1:200 FcR blocking reagent (Miltenyi Biotec, Bergisch Gladbach, D) for 10 min at 4 °C. After blocking, the cells were stained with 1:100 CD14-PE/Cyanine 7 (clone 63D3; BioLegend, San Diego, Ca, USA) or with the following antibodies at either 1:100 (1 x) or 1:10 (10 x): ACK1-FITC (#ACK1-FITC; polyclonal, FabGennix, Frisco, TX, USA); CLEC5A-PE (clone 283834; R&D Systems, Minneapolis, MN, USA); Lox1-APC (clone 15C4; BioLegend, San Diego, CA, USA); and FLNB-Alexa Fluor 680 (#ABIN5002742, polyclonal, Antibodies-online, Aachen, G). The cells were incubated for 30 min on ice. After the cells were blocked with FcR, unstained cells were used as controls. The samples were washed and resuspended in flow buffer before they were analyzed on an LSRII (BD Biosciences) instrument equipped with the following wavelength lasers: 405 nm, 488 nm, 561 nm, and 639 nm. A total of 10 000 events were recorded for each sample. After exclusion of debris and cell doublets, unstained cells were used to define gates for cells with positive expression of the factors of interest. The percentage of positive cells and the mean fluorescence intensity of the factors of interest in CD14^+^ monocytes were subsequently correlated with the resorption levels of the differentiated cells. Linear regression models were used to determine significance.

## Data Availability

RNA-seq data generated from eight human donors at Days 0, 2, 5, and 9 of osteoclast differentiation in vitro have been deposited in the Gene Expression Omnibus (GEO) database under accession code GSE246769. Processed scRNA-seq data are available at the open science framework: https://osf.io/9xys4/. Expression data from iliac crest biopsies of healthy and osteoporotic subjects are available at ArrayExpress under E-MEXP-1618 (ref.^29^), from peripheral blood monocytes of subjects with low and high BMD at GEO GSE56815 (ref.^31,32^), from the fracture time course in mice at GSE152677 (ref.^34^), and from human osteoclasts before and after differentiation at GSE225974 (ref.^20^). The processed sequencing data, metadata, and scripts necessary to recapitulate the analyses are available at the open science framework: https://osf.io/9xys4/and at GitHub: https://github.com/drarauch/BulkOsteoclasts.
